# Stress-Responsive Periplasmic Chaperones in Bacteria

**DOI:** 10.3389/fmolb.2021.678697

**Published:** 2021-05-11

**Authors:** Hyunhee Kim, Kevin Wu, Changhan Lee

**Affiliations:** ^1^Department of Biological Sciences, Ajou University, Suwon, South Korea; ^2^Molecular, Cellular, and Developmental Biology, Howard Hughes Medical Institute, University of Michigan, Ann Arbor, MI, United States; ^3^Department of Biophysics, University of Michigan, Ann Arbor, MI, United States

**Keywords:** ** periplasmic** chaperone, Spy, DegP, HdeA, HdeB, UgpB

## Abstract

Periplasmic proteins are involved in a wide range of bacterial functions, including motility, biofilm formation, sensing environmental cues, and small-molecule transport. In addition, a wide range of outer membrane proteins and proteins that are secreted into the media must travel through the periplasm to reach their final destinations. Since the porous outer membrane allows for the free diffusion of small molecules, periplasmic proteins and those that travel through this compartment are more vulnerable to external environmental changes, including those that result in protein unfolding, than cytoplasmic proteins are. To enable bacterial survival under various stress conditions, a robust protein quality control system is required in the periplasm. In this review, we focus on several periplasmic chaperones that are stress responsive, including Spy, which responds to envelope-stress, DegP, which responds to temperature to modulate chaperone/protease activity, HdeA and HdeB, which respond to acid stress, and UgpB, which functions as a bile-responsive chaperone.

## Introduction

Proteins are involved in various cellular pathways, such as replication of DNA, gene regulation and metabolism, in all living organisms. Therefore, the protein quality control in bacteria is directly associated with bacterial survival in various natural environments and host niches. Bacterial protein synthesis and homeostasis are targeted by natural and man-made antimicrobial substances (McCoy et al., 2011; Klebanoff et al., 2013; Bednarska et al., 2016). In order to cope with these damaging conditions, bacteria need to immediately sense environmental changes and react rapidly and appropriately. Some types of stressors, such as temperature change, desiccation, and acidity, have negative impacts on protein stability.

Molecular chaperones are key players of protein folding homeostasis (proteostasis). A sophisticated network of chaperones exists in every organism, and these chaperones fulfill various roles, including preventing protein aggregation, disaggregating aggregated proteins, and aiding in protein folding (Tyedmers et al., 2010; Hartl et al., 2011; Valastyan and Lindquist, 2014). Bacterial chaperone systems have been extensively studied in *Escherichia coli*. In the cytoplasm of *E. coli*, the DnaK (Hsp70)/DnaJ (Hsp40)/GrpE and GroEL/GroES chaperone systems function as foldases in an ATP-dependent manner, and they aid in the folding of newly translated proteins and unfolded proteins (Saibil, 2013). Under stress conditions such as heat shock, the small heat shock proteins (sHsps) IbpA and IbpB act as holdases that serve as transient reservoirs for the prevention of irreversible aggregation and the facilitation of aggregated-protein resolubilization by disaggregating chaperones, which occurs in an ATP-independent manner (Mogk et al., 2019). Subsequently, DnaK/DnaJ/GrpE and the ClpB (Hsp100) disaggregase cooperatively interact with and unfold protein aggregates (Mogk et al., 2018).

**FIGURE 1:**
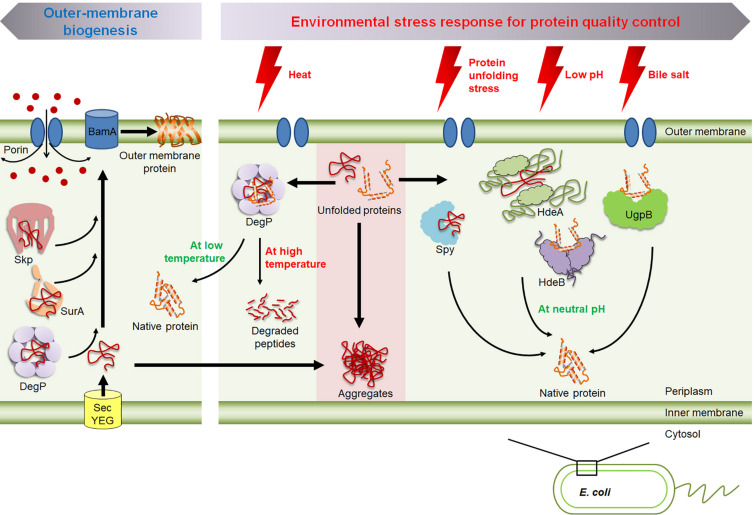
An overview of the molecular chaperones in the periplasm of *E. coli.* Skp, SurA, and DegP are involved in the biogenesis of outer membrane proteins; i.e., preventing their aggregation and facilitating their insertion into the membrane. The shift of temperature modulates DegP’s chaperone and protease activity. HdeA and HdeB prevent acid-induced protein aggregation. Spy and UgpB respond to envelope stress and bile stress, respectively.

Gram negative bacteria possess a periplasmic space that is located between the inner cytoplasmic membrane and the bacterial outer membrane. A number of proteins with diverse function are present in the periplasm, for example, degradative enzymes such as phosphatases, proteases, and endonucleases; and antibiotic detoxifying enzymes such as β-lactamases, alkyl sulfodehydrases, and aminoglycoside phosphorylating enzymes; binding proteins for amino acids, sugars and vitamins (Neu and Heppel, 1965; Han et al., 2014). A wide range of outer membrane proteins and proteins that are secreted into the external cellular region must pass through the periplasm (Szewczyk and Collet, 2016). SecYEG complex mediates the transports of the most precursor proteins across the inner membrane (Van Den Berg et al., 2004). The precursor protein is unstructured and its signal sequence is cleaved during the translocation (Van Den Berg et al., 2004). After translocation, proteins are on the different folding pathways, and Skp and SurA are major periplasmic chaperones which can bind to the variety of unfolded outer membrane proteins during the transit through the periplasm, preventing their aggregation and facilitating their insertion into the membrane (Stull et al., 2018a; Figure 1). The outer membrane protein assembly factor BamA facilitates folding of the chaperone-bound outer membrane proteins into lipid bilayers (Bennion et al., 2010; Patel and Kleinschmidt, 2013). Homotrimeric Skp is a functional form as a chaperone and has a “jellyfish”-like structure with three α-helical flexible tentacles which provide a hydrophobic cavity inside to accommodate a client protein (Schiffrin et al., 2016). SurA has three domains consisting of core domain and two peptidylprolyl isomerase domains, and recent study has shown that a client protein can bind to a cradle formed between the SurA domains (Calabrese et al., 2020).

Periplasmic proteins are more exposed to external environmental stresses than cytoplasmic proteins are, as porins in the outer membrane allows for the free diffusion of small molecules below ∼600 Da (Nikaido, 2003; Figure 1). Thus, periplasmic proteins must be able to cope with extreme environmental changes. For example, as enteric bacteria pass through the host digestive system, the bacteria encounter various host-defense barriers that target protein stability, including gastric acid in the stomach and bile in the duodenum, both of which denature proteins (Dill and Shortle, 1991; Cremers et al., 2014). Therefore, various periplasmic chaperones are transcriptionally and/or post-translationally regulated under stress conditions. In this review, we discuss how these stress-responsive periplasmic chaperones respond to environmental stresses and modulate their activity, but we do not discuss the complete set of stress-responsive periplasmic chaperones, as they have recently been comprehensively reviewed (Stull et al., 2018a). Instead, we have chosen to focus particularly on the periplasmic chaperones which are regulated at the post-translation level by environmental stresses such as the temperature-responsive chaperone/protease DegP; the acid-responsive chaperone, HdeA and HdeB; and the bile-responsive chaperone, UgpB. We also discuss Spy which is nearly not expressed under non-stress conditions, but it is massively induced upon exposure to the envelope stress.

## Distinct Nature in the Periplasm for Chaperones Compared to the Cytoplasm

The folding environment in the periplasmic space is different from that in the cytoplasm. For example, ATP is absent in the periplasm. Many cytoplasmic chaperones utilize ATP to modulate chaperone activity (Hartl et al., 2011). Since ATP is absent in the periplasm, periplasmic chaperones require a different means of modulating their activity. Another difference between the periplasm and the cytoplasm is the thiol-disulfide redox state. In the periplasm, proteins have high potential for forming disulfide bond, which is not simply due to the presence of oxygen. The oxidizing environment directly results from the presence of the disulfide bond (Dsb) family of enzymes in the periplasm. Dsb proteins catalyze disulfide bond formation and isomerization (Bardwell, 1994; Ito and Inaba, 2008). Deleting the genes encoding the Dsb proteins dramatically reduces the abundance of a range of proteins that normally contain disulfides due to the effect of disulfides on protein folding and stability (Bardwell et al., 1991).

## Stress-Responsive Chaperones in Periplasm

The bacterial envelope and periplasm are at the front lines of external stress. Because of this, bacteria have several pathways to sense and respond to these stresses. In *E. coli*, five response pathways, designated BaeSR two component system, CpxAR-two component system, phase shock protein (Psp) system, regulator of capsular synthesis (Rcs) system, and sigma factor σ^*E*^, are responsible for responding to envelope and periplasmic stresses (Bury-Mone et al., 2009). Periplasmic chaperones are regulated by these stress-responsive pathways. σ^*E*^ responds to heat, membrane, and periplasmic stresses, including those induced by alcohol species and detergents (Ades et al., 2003). The Skp and SurA chaperones are regulated by σ^*E*^, as is the protease/chaperone DegP, the prolyl isomerase FkpA, and the disulfide oxidoreductase DsbC (Rhodius et al., 2006; Sklar et al., 2007; Bury-Mone et al., 2009; Stull et al., 2018a). DegP and FkpA are also regulated by the Cpx pathway (Bury-Mone et al., 2009). The envelope-stress responsive chaperone Spy is induced by the Bae, Cpx, and Rcs pathways (Bury-Mone et al., 2009; Quan et al., 2011). The periplasmic chaperones OsmY and Ivy are under the control of the Rcs pathway (Bury-Mone et al., 2009; Lennon et al., 2015). In addition to being transcriptionally regulated, periplasmic chaperones, including HdeA, HdeB and UgpB, are also regulated at the post-translational level (Foit et al., 2013; Lee et al., 2020). Thus, in summary, the vast majority of periplasmic chaperones are under some mode of stress control, a fact that has long been known to be true for chaperones present in other compartments, particularly the cytoplasm (Richter et al., 2010).

## The Envelope-Stress Responsive Chaperone, Spy

Spy (Spheroplast Protein Y) was initially characterized as a protein whose expression is very strongly increased by spheroplast formation, hence its name (Hagenmaier et al., 1997). Spy is very weakly expressed in unstressed cells, but it is massively induced by spheroplasting, a process that involves disrupting the outer bacterial envelope using treatments such as lysozyme and EDTA (Hagenmaier et al., 1997). That Spy functions as a chaperone was discovered by employing a genetic selection system that uses a protein-folding sensor linking protein folding to antibiotic resistance (Quan et al., 2011). The protein folding sensor is a tripartite fusions between two proteins, in which an unstable test protein is inserted into the middle of a selectable antibiotic marker (Foit et al., 2009). The protein-folding sensors used in the discovery of Spy, UgpB and other proteins exhibiting chaperone activity (Foit et al., 2009; Lennon et al., 2015; Lee et al., 2020). For the selection system used to discover Spy, an unstable variant of immunity protein 7 (Im7) was inserted between two halves of β-lactamase, which confers penicillin resistance (Quan et al., 2011). This makes antibiotic resistance dependent on the folding of the unstable test protein. It was found that overexpression of Spy acts to stabilize the Im7-fused protein-folding sensor and consequently confers high levels of antibiotic resistance to *E. coli* (Quan et al., 2011). The mechanism underlying Spy’s chaperone function has subsequently been very well characterized (Quan et al., 2011; Horowitz et al., 2016; Koldewey et al., 2016; Stull et al., 2016; Wu et al., 2019; Mitra et al., 2021).

Spy is a small protein (16 kDa) which forms a stable dimer in solution (Quan et al., 2011). Each Spy monomer contains four α-helices. The dimeric Spy forms a cradle-like structure through an antiparallel coiled-coil interaction. The concave surface of Spy is dominated by positive charges with two hydrophobic patches located in the bottom of cradle (Kwon et al., 2010; Quan et al., 2011). Studies have shown that Spy used almost the entire interior of cradle to rapidly recognize its client proteins, and thereby effectively preventing protein aggregation under stress conditions (Quan et al., 2011; Koldewey et al., 2016).

**FIGURE 2:**
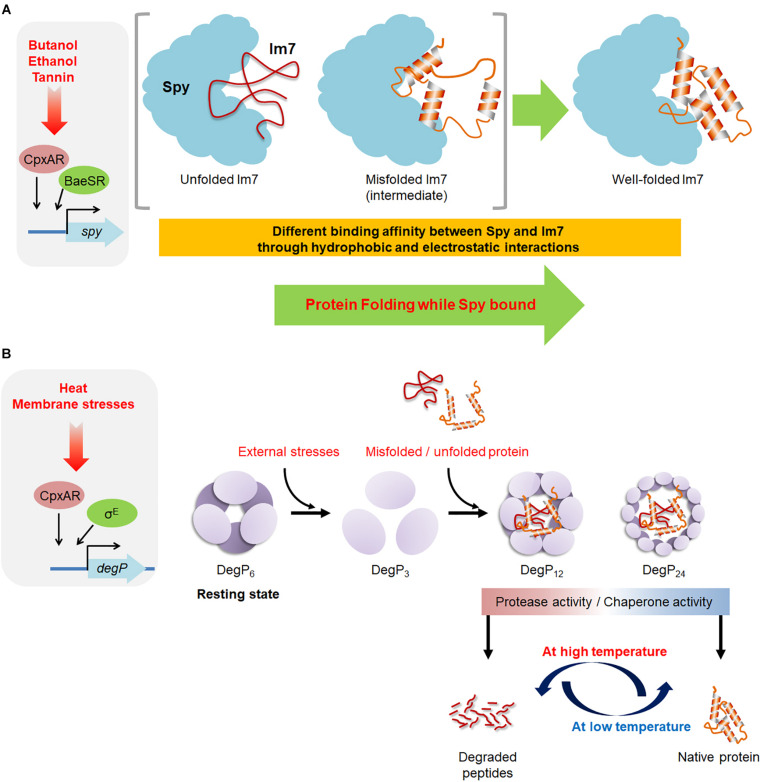
An overview of the molecular mechanisms of Spy and DegP. **(A)** The envelope-stress conditions occurring under butanol, ethanol, and tannin exposure induce the production of the chaperone Spy, which binds to misfolded or unfolded substrates by electrostatic and hydrophobic interactions. Spy prevents further aggregation of the substrate and enables proper folding of the substrate while it is bound to Spy. **(B)** DegP exhibits both the chaperone and protease activity. The hexameric form of DegP is a resting state. The exposure to external stresses, the hexameric DegP presumably dissociated to trimers, which are a basic building unit for the formation of large cage-like structures. The 12- and 24-mer of cage-like DegP complexes can encapsulate bound substrates for its chaperone and protease activity. The stress-induced conformation changes of DegP and the folding state of the bound substrates are thought to be involved in determining the fate of DegP as a chaperone or protease.

The expression of Spy is tightly repressed under non-stress conditions but is strongly induced by spheroplasting treatments (Hagenmaier et al., 1997) and the protein denaturants butanol, ethanol, and tannin (Figure 2A; Quan et al., 2011). Spy is so strongly induced after tannin and butanol treatments that it can comprise up to 25% of the total periplasmic protein content (Quan et al., 2011). Spy expression is controlled by the CpxAR and BaeSR two-component signal transduction systems, which respond to protein-unfolding stress in the cellular envelope (Raivio et al., 2000; Raffa and Raivio, 2002; Bury-Mone et al., 2009). Constitutive *baeSR* mutants greatly overproduce the Spy protein (Quan et al., 2011). This rapid and massive production of Spy under stress is clearly an important way to modulate the chaperone function of Spy in the periplasm. Spy deletion mutants are sensitive to tannin and zinc treatments (Quan et al., 2011; Wang and Fierke, 2013). In a recent study, Spy was isolated as suppressor in *elyC* mutants at low temperature (21°C) (Kouidmi et al., 2018). ElyC is a factor involved in peptidoglycan biosynthesis at low temperatures (Paradis-Bleau et al., 2014), The deletion of *elyC* gene increases the amount of cellular protein aggregates, and the overexpression of Spy can significantly reduce the amount of protein aggregates and completely suppress the defect in peptidoglycan biosynthesis, suggesting that the absence of ElyC causes protein folding problem in the periplasm (Kouidmi et al., 2018). Spy homologs are widely present in enterobacteria and proteobacteria, and in some cyanobacteria (Quan et al., 2011).

The mechanism through which Spy acts on its client proteins has been extensively investigated, particularly with the model substrate, Im7 (Figure 2A). The prevailing paradigm has been that hydrophobic interactions are the major driving forces leading chaperones to recognize its clients (Kim et al., 2013). However, it has recently been shown that electrostatic interactions also play an important role in chaperone actions, particularly for Spy (Coyle et al., 1997; Koldewey et al., 2016, 2017; Lee et al., 2018). These results are consistent with the recent observation that Super Spy variants with enhanced chaperone activity not only exhibit increased hydrophobicity but also an increase in electrostatic interactions (He et al., 2020). Electrostatic interactions are effective over much longer distances than hydrophobic interactions (Selzer and Schreiber, 1999; Schreiber et al., 2009). The highly basic nature of Spy’s substrate-binding surface drives charge-charge interactions with client proteins (Koldewey et al., 2016). These long-range electrostatic interactions between the positively charged Spy and its negatively charged client proteins increase the association rate between these two entities, allowing Spy to rapidly bind to unfolded, aggregation-prone substrates, thereby preventing their aggregation (Koldewey et al., 2016). After the substrate has been recognized via long-range electrostatic interactions, short-range hydrophobic interactions occur between Spy’s central cradle region and its unfolded substrates, resulting in the stabilization of the Spy-substrate complex (Koldewey et al., 2016).

Following studies showed that two folding model substrates, Im7 and Fyn SH3, are allowed to fold into their native states while they are bound to Spy (Stull et al., 2016; Wu et al., 2019). Protein folding while bound to chaperone Spy is apparently dependent on the affinity between chaperone and its client (Wu et al., 2019). The more tightly Spy binds its clients, the more it slows the folding of bound clients. Evolution seems to achieve this delicate chaperone action by fine-tuning Spy’s binding affinity to its clients (Wu et al., 2019). By having too strong binding affinity, Spy could unfold its client proteins and cause toxic effects on cells; while having too weak affinity would sacrifice its chaperone activity (Wu et al., 2019). Thus, having a modest binding affinity might be a better evolutionary solution for chaperone, like Spy, to mediate protein folding before more advanced regulatory mechanisms were developed. Upon substrate folding, the hydrophobic contacts between Spy and its substrates are reduced, increasing the dissociation rate and promoting the substrate release (Koldewey et al., 2016; Stull et al., 2016). A very recent study showed that the chaperone mechanism of Spy could be substrate-dependent (Mitra et al., 2021). In the case of apo-flavodoxin, Spy can rapidly recognize and stabilize a partially unfolded state, and thereby effectively suppressing protein aggregation during stresses. On the other hand, tight substrate binding eliminates the possibility for apo-flavodoxin to refold to its native state while bound to Spy. This study highlights the importance of substrate-dependent chaperone mechanisms, in which chaperones could have different modes of action for different client proteins (Table 1; Koldewey et al., 2017). Yet, future studies are required to demonstrate how these client proteins which are tightly bound to Spy are subsequently released to the solution as the stresses are removed.

**TABLE 1:** Various chaperone actions and mechanisms of Spy.

*In vitro* client	Spy’s action	References
Malate dehydrogenase (MDH)	Suppress protein aggregation during stresses	Quan et al., 2011
Aldolase	Suppress protein aggregation during stresses	Quan et al., 2011
DsbB	Suppress protein aggregation during stresses	Quan et al., 2011
Alkaline phosphatase	Suppress protein aggregation during stresses	Quan et al., 2011
α-lactalbumin (α-LA)	Suppress protein aggregation during stresses	He et al., 2020
Im7	Allow the client to refold to its native state while bound	Stull et al., 2016
Fyn SH3	Allow the client to refold to its native state while bound	Wu et al., 2019
Apo-flavodoxin	Inhibit the client to refold to its native state	Mitra et al., 2021

## The Temperature-Responsive Protease/Chaperone, DegP

DegP (also called HtrA or protease Do) is known to play a central role in the protein quality control network in the periplasm through its dual function; i.e., its protease activity for the removal of misfolded or damaged proteins and its chaperone activity. DegP was first identified as a protease essential for growth of *E. coli* at high temperature (Lipinska et al., 1989; Strauch et al., 1989). DegP is highly conserved in most Gram-negative bacteria (Lipinska et al., 1990; Pallen and Wren, 1997). *DegP* deletion mutant is lethal at high temperature (42°C) (Skorko-Glonek et al., 1995). Of note, overexpression of protease-deficient forms of DegP can sufficiently suppress the lethal phenotype, suggesting that DegP protease activity is not mandatory for heat tolerance (Misra et al., 2000; CastilloKeller and Misra, 2003). DegP is part of a large serine proteases-related family which is found in most organisms (Chang, 2016) and is upregulated by the σE and Cpx pathway under heat, membrane, and periplasmic stresses (Danese et al., 1995; Alba and Gross, 2004). DegP is also associated with thermal, osmotic and oxidative stress tolerance (Pallen and Wren, 1997; Gunasekera et al., 2008). Moreover, *degP* deletion mutants of several pathogenic bacteria are attenuated, suggesting that DegP might be involved in bacterial virulence (Pallen and Wren, 1997).

The mature DegP protein is composed of three domains, the chymotrypsin-related protease domain which contains an active site His-Asp-Ser motif at the N terminus and the two PDZ domains (PDZ1 and PDZ2) at the C terminus which play important roles in substrate recognition as well as in the transformation of DegP to large cage-like structures (Ortega et al., 2009). Purified DegP present as a hexamer in solution and is composed of a dimer of two trimers, which is a resting state of DegP (Figure 2B; Krojer et al., 2002; Clausen et al., 2011). The hexameric DegP can be activated through the rearrangement into 12-mers/24-mers of cage-like structure, which can encapsulate substrate proteins (Krojer et al., 2002, 2008; Jiang et al., 2008; Kim et al., 2011). The trimeric DegP is a building unit for the different large cage-like structure and thus it is assumed that DegP undergoes a large structural changes from the hexameric resting state through a trimeric state to the higher oligomeric state (Li et al., 2014; Thompson et al., 2014). The presence of the substrate triggers the reorganization of hexameric DegP into the large cage-like structure (Jiang et al., 2008; Krojer et al., 2008).

Of note, at low temperature (below 28°C), the protease activity drops and DegP mostly function as a chaperone, suggesting that DegP switches from chaperone to proteolytic activity as a function of temperature (Spiess et al., 1999). However, the underlying molecular mechanism by which DegP switches from chaperone to protease upon temperature shift is still not fully understood. One hypothesis that a temperature-induced conformational change occurs in the proteolytic site (Krojer et al., 2002; Ortega et al., 2009). At low temperatures, the proteolytic serine (Ser-210) residue is present in an inactive conformation away from other residues of the catalytic triad, resulting that only chaperone activity is exhibited. However, it has been suggested that the elevation of temperature may induce conformational change in Ser-210 to assemble functional catalytic triad, thus exhibiting protease activity. In terms of folding state of substrates, DegP degrades unfolded outer membrane proteins but stabilizes folded outer membrane proteins (Krojer et al., 2008), suggesting the folding state of bound substrates determines the function of DegP. Temperature undoubtedly affects the folding state of proteins; thus these results partially explain the role of temperature in functional transition of DegP. In recent study, the analysis of the interaction and dynamics of the PDZ-domains of DegP by high-resolution NMR spectroscopy reveals that PDZ1-PDZ2 interaction through Met-280 and Tyr-444 residues is crucial for the temperature-dependent regulation in the oligomeric states of DegP (Šulskis et al., 2020).

As a molecular chaperone, DegP enhances the *in vitro* refolding of the *E. coli* periplasmic α-amylase MalS and citrate synthase at low temperature (Spiess et al., 1999). In this study, deletion of the PDZ domains did not affect refolding yields, suggesting that the protease domain itself has chaperone activity (Spiess et al., 1999). DegP has also been shown to prevent aggregation of heat-denatured citrate synthase and lysozyme by acting as a holdase (Skorko-Glonek et al., 2007). DegP is also involved in the biogenesis of outer membrane proteins by protecting them from aggregation and proteolytic degradation during their transport across the periplasm (Krojer et al., 2008). Deletion of *degP* shows a synthetically lethal phenotype with deletion of either a *surA* or *skp*, suggesting that DegP plays a role in outer membrane protein biogenesis as SurA and Skp (Sklar et al., 2007).

## The Acid-Responsive Chaperones, HdeA and HdeB

*Escherichia coli* has two acid-responsive periplasmic chaperones, HdeA and HdeB. HdeA and HdeB are very small proteins, 11 and 12 kDa, respectively, and they share 17% amino acid sequence identity and have similar structures (Wang et al., 2012; Stull et al., 2018a). Both proteins are well-folded α-helical dimers at neutral pH that bury their hydrophobic surfaces in their dimerization regions (Yang et al., 1998). It appears that HdeA functions at low pH levels (pH 1–3), whereas HdeB functions under mildly acidic conditions (pH 4–5) (Kern et al., 2007; Malki et al., 2008; Dahl et al., 2015; Ding et al., 2015). The genes encoding the HdeA and HdeB proteins form an operon that is located on a genomic island (termed as an acid fitness island) (Mates et al., 2007). The *hdeAB* operon was also identified in *Shigella flexneri* and *Brucella abortus* (Hong et al., 2012). The transcription of *hdeAB* is induced by the overproduction of the DNA-binding transcriptional regulator YdeO, which is upregulated by the acid-responsive EvgSA two component system (Masuda and Church, 2003), and the RNA polymerase holoenzyme assembly factor Crl can also increase *hdeAB* expression through RpoS (Dudin et al., 2013). HdeA is the 6th most abundant protein in the cell during stationary phase (Link et al., 1997). Thus, bacteria can immediately respond to acid stress.

Under acidic conditions, the acid stress causes protein misfolding by disrupting the hydrogen bonds and salt bridges required for protein folding. Because of the porous nature of the outer membranes of Gram-negative bacteria, changes in the pH of the surrounding environment cause a corresponding rapid change in the pH of the periplasmic space. It was recently shown that a large drop in extracellular pH triggers a surge in periplasmic chloride ions to a concentration that can exceed 0.6 M due to the Donnan effect (Stull et al., 2018b). In the low pH, the increase of chloride anions accelerates protein aggregation in the periplasm, because it neutralizes the positive charges of the protein, minimizing the force of the electrostatic repulsion between unfolded proteins, that would prevent protein aggregation (Stull et al., 2018b). When bacteria transit through the acidic environment of the host stomach, protecting their periplasmic proteins from acid stress is necessary for bacterial survival. Consistently, an *hdeA* mutant showed reduced survival after exposure to low pH conditions (Waterman and Small, 1996; Gajiwala and Burley, 2000; Mates et al., 2007).

**FIGURE 3:**
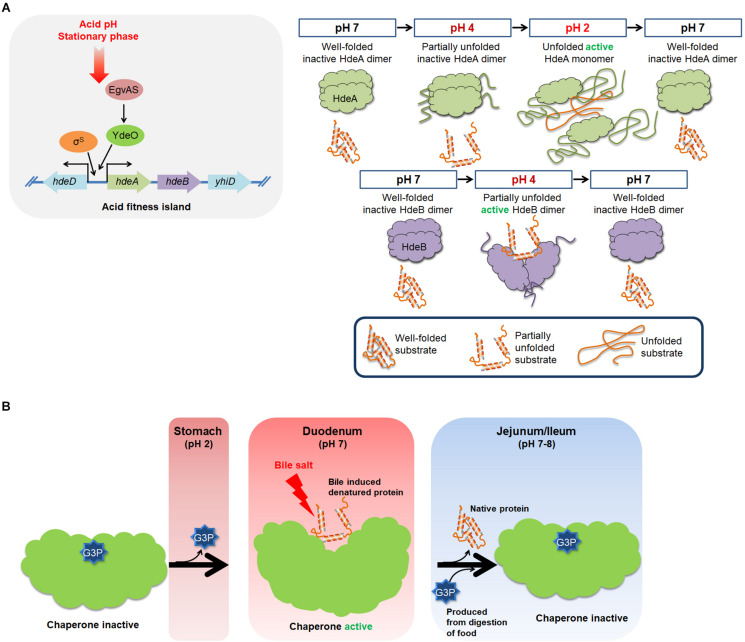
An overview of the molecular mechanism of HdeA, HdeB, and UgpB. **(A)** Acidic pH levels and stationary phase can induce the expression of HdeA and HdeB, which act to protect a broad range of periplasmic proteins from acid-induced aggregation. At neutral pH, HdeA exists as a well-folded homodimer in an inactive state. At pH 2, the unfolded monomer form of HdeA can bind to the hydrophobic surfaces of denatured substrates and protect them from aggregation. HdeB also protects proteins from acid-induced aggregation. In contrast to HdeA, HdeB exhibits chaperone activity at pH 4 in a dimeric, partially unfolded form. **(B)** The *E. coli* periplasmic G3P-binding protein UgpB exhibits chaperone activity against bile salts. The chaperone activity of UgpB is evident only when G3P is stripped off of UgpB. Bacterial cells must pass through the acidic stomach and the bile-rich duodenum to reach the lower intestine where they colonize. Acidic pH levels in the stomach can unfold UgpB, resulting in the dissociation of G3P. Consequently, UgpB exerts chaperone activity to prevent bile salt-induced protein aggregation in the duodenum. Release of G3P exposes the core cleft region of UgpB, which functions as a chaperone active surface. An increase in the G3P concentration in the jejunum and ileum resulting from the digestion of food triggers a functional transition of UgpB from molecular chaperone to G3P transporter.

To function as chaperones, HdeA and HdeB appear to utilize changes in external pH to trigger chaperone activation, inactivation, and substrate-protein refolding (Figure 3A). Upon exposure to acidic conditions, HdeA and HdeB partially unfold, resulting in the activation of their chaperone activities (Foit et al., 2013; Dahl et al., 2015; Yu et al., 2019). HdeA undergoes a dramatic conformational change from a well-folded chaperone-inactive dimer to a partially disordered, chaperone-active monomer (Yang et al., 1998; Foit et al., 2013; Salmon et al., 2018). The hydrophobic surfaces involved in the dimeric interaction surface become exposed and serve as a substrate-binding site (Yu et al., 2019). Recent NMR studies have proposed that HdeA contains two hydrophobic patches (site I: 49–55AA, site II: 28–35AA) that are involved in client binding and three acid-sensitive regions, A, B, and C (A: 46–51 AA; B: 34–40 AA; and C: 24–29 AA), that act as structural triggers that regulate the exposure of the two client-binding sites (Yu et al., 2019). Thus, multiple steps occur in the HdeA activation mechanism during the transition to a low pH (Yu et al., 2019). Upon returning to neutral pH, HdeA-substrate complexes spontaneously dissociate, and the substrates are released in a folding-competent state (Tapley et al., 2010). Thus, the cycle of chaperone action for HdeA is intricately modulated by host-induced pH changes.

In HdeA, client-binding site I is located in a relatively peripheral region of the HdeA dimer structure, where it is shielded by the N-terminal segment of the other HdeA monomer (Figure 3A; Yu et al., 2019). The acidic residues in acid-sensitive region A (Glu-46, Asp-47, and Asp-51 with pKa values of 4.07, 4.14, and 3.83, respectively) are deprotonated under neutral and near-neutral conditions (pH > 4), ensuring an electrostatic interaction with the N-terminal region of HdeA (Garrison and Crowhurst, 2014). At pH values below 4, the protonation of these residues disrupts the electrostatic interaction with the N-terminal region, exposing client-binding site I (Yu et al., 2019). The regulatory role of the N-terminal region is supported by the observation that an HdeA variant containing a nine residue N-terminal deletion shows enhanced interaction with its client proteins (Gajiwala and Burley, 2000) and also exhibits partial anti-aggregation activity at pH 4.0, whereas wild-type HdeA is inactive at this pH (Dahl et al., 2015; Yu et al., 2019). A constitutively active mutation in HdeA (D20A/D51A) may also affect the regulatory function of the N-terminal region (Foit et al., 2013). Client-binding site II is tightly packed in the structural core of the HdeA dimer and can only be exposed after extensive disruption of the dimeric interface. At pH values above 4, the two acid-sensitive regions B and C stabilize the dimer interface via inter- and intrasubunit contacts (Yu et al., 2019). As the pH decreases to values below 4, local structural destabilization disrupts the interactions between acid-sensitive regions B and C, partially exposing client-binding site II (Yu et al., 2019). Further decreases in pH to values below 2 lead to a complete collapse of the protein structure of HdeA, resulting in a fully active chaperone (Yu et al., 2019).

The periplasmic chaperones SurA and DegP have been implicated as HdeA substrates under low-pH conditions (Zhang et al., 2011), and HdeA suppresses the acid-induced aggregation of SurA *in vitro* (Zhang et al., 2011). In addition, SurA or DegP assists HdeA in refolding acid-denatured alkaline phosphatase *in vitro*, suggesting that HdeA protects chaperones, such as SurA and DegP, and subsequently enables these chaperones to participate in the refolding of substrate proteins that are dissociated from HdeA following the transition to neutral pH (Zhang et al., 2011). Fibrillation of HdeA at pH 2 has been observed in a recent study, and these fibrils can be resolubilized following a shift to pH 7 (Miyawaki et al., 2019). This unusual reversibility of fibrillation for HdeA suggests this is another pH-dependent regulatory mechanism for HdeA.

The functional mechanism of HdeB is less well understood than that of HdeA. In contrast to HdeA, HdeB appears to function as a dimer in preventing acid-induced protein aggregation and facilitating refolding upon neutralization (Dahl et al., 2015). HdeB exhibits optimal chaperone activity at pH 4 *in vitro* (Dahl et al., 2015). HdeB has a well-folded dimeric structure at neutral pH, but it starts to undergo partial unfolding near pH 3 and reaches an overall unfolded state at pH 2–1.5 (Dahl et al., 2015; Ding et al., 2015). HdeB does not show significant chaperone activity at pH 2 (Dahl et al., 2015), suggesting that the activation of the HdeB chaperone function is linked to pH-dependent conformational changes rather than monomerization (Ding et al., 2015).

A protein sequence alignment of the HdeA and HdeB homologs revealed that the N-terminal nine residues present in HdeA are lacking in the N-terminus of HdeB (Yu et al., 2019). Since these residues are involved in protecting the client-binding site on HdeA, the client-binding site in HdeB is thus presumably constitutively exposed, allowing HdeB to interact with its client proteins under non-acidic conditions without the need for protein unfolding (Yu et al., 2019). Consistent with this hypothesis, HdeB prevents the aggregation of some substrates (like lactate dehydrogenase) at pH 7.5 *in vitro* (Lennon et al., 2015). HdeB copurifies with HdeA (Arnqvist et al., 1994), but HdeB and HdeA do not appear to heterodimerize *in vitro* (Kern et al., 2007; Dahl et al., 2015).

## The Bile-Responsive Chaperone, UgpB

UgpB is a periplasmic substrate-binding protein and a component of the uptake of glycerol phosphate system (Ugp system), which is also known as the glycerol-3-phosphate (G3P) ATP-dependent binding cassette transporter. It is conserved in various Gram-negative bacteria, including *E. coli* (Argast and Boos, 1979; Wuttge et al., 2012). Purified UgpB binds to G3P and glycerophosphocholine *in vitro* (Wuttge et al., 2012). These bound substrates are transferred to the inner membrane associated with the Ugp complex and then transported into the cytosol through the hydrolysis of ATP (Wuttge et al., 2012). Transported G3P can be utilized as a carbon or phosphate source (Wanner, 1996). We have recently shown that UgpB also functions as a bile-responsive chaperone (Lee et al., 2020). Bile is an amphipathic compound that assists mammals in the absorption of lipids (Heaton, 1969; Gunn, 2000; Urdaneta and Casadesus, 2017). Bile also exhibits potent antimicrobial activity mediated by its ability to disrupt cell membranes, cause DNA damage, and, importantly in reference to its chaperone activity, cause protein unfolding and aggregation (Prieto et al., 2004; Merritt and Donaldson, 2009; Cremers et al., 2014; Urdaneta and Casadesus, 2017). Bile enters the bacterial cytosol through a flip-flop mechanism (Cabral et al., 1987), and its entry leads to the induction of various chaperones, including Hsp33, DnaK, and GroEL (Flahaut et al., 1996; Bernstein et al., 1999; Leverrier et al., 2003; Ruiz et al., 2013; Cremers et al., 2014). The bacterial periplasm is presumably more highly exposed to bile than the cytosol is, owing to the porous nature of the outer periplasmic membrane, but, surprisingly, very little is known about how periplasmic proteins are protected against bile.

High-throughput transposon sequencing (Tn-Seq), in combination with a periplasmic protein-folding sensor, allowed us to establish that UgpB has chaperone activity (Lee et al., 2020). Tn-Seq allows one to compare the transposon-insertion frequencies between all genes in the genomes of two populations, one of which has been subject to genetic selection (Van Opijnen et al., 2009; Burby et al., 2017). Gene disruption by transposon insertion may affect the growth rate under the applied selection condition, and consequently certain genes shows altered transposon insertion frequencies. A periplasmic protein-folding sensor, which links protein stability to antibiotic resistance, provides powerful selection power for Tn-Seq. The unstable Im7 variant was used as a test protein in the context of the protein-folding sensor (Quan et al., 2011; Lee et al., 2020). The gene encoding Spy was rediscovered using this method (Lee et al., 2020). Another locus that had a highly elevated transposon insertion frequency was the *pstSCA* operon (Lee et al., 2020). *PstSCA* encodes an ATP-binding cassette transporter for phosphate uptake, and the disruption of each of these genes alters the expression levels of downstream genes, including *ugpB* (Gardner et al., 2015). In *pst* mutants, UgpB becomes the most abundant protein in the periplasm, and overexpression of UgpB acts to stabilize the protein-folding sensor *in vivo* (Lee et al., 2020).

**FIGURE 4:**
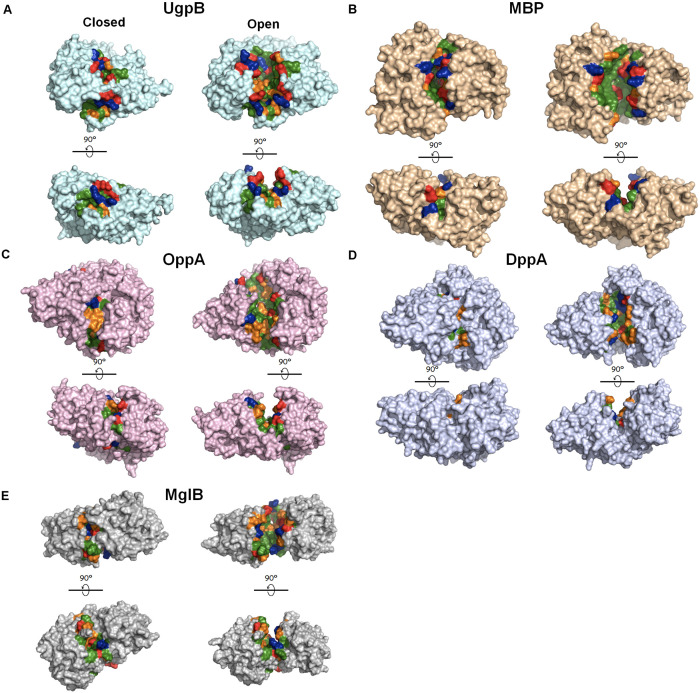
Crystal structures of periplasmic substrate-binding proteins with open and closed conformations. The ligand-free open conformations and ligand-bound closed conformations are shown on the right and left, respectively, for each panel. Upon the release of the ligand, the buried core region is exposed to the solvent. The solvent–exposed cavities in the open conformation were detected using the CASTp 3.0 server (Tian et al., 2018) and were highlighted according to the characteristics of the residues (green, hydrophobic residues; orange, polar residues; blue: positively charged residues; red: negatively charge residues. **(A)** *E. coli* UgpB (pdb: 6 × 84 [open] and 4aq4 [closed]); **(B)** *E. coli* MBP (MalE, pdb: 1omp [open] and 1anf [closed]); **(C)** *E. coli* OppA (pdb: 3tch [open] and 3tcg [closed]); **(D)** *Pseudoalteromonas sp.* DppA (pdb: 4qfk [open] and 4qfn [closed]); **(E)** *Treponema pallidum* MglB (pdb: 6bgd [open] and 6bgc [closed]). Figures were made in pymol.

Bile induces UgpB expression. Disrupting the *ugpB* gene confers bile sensitivity (Nichols et al., 2011; Hernandez et al., 2012), and overexpressing UgpB confers bile resistance (Lee et al., 2020). UgpB can prevent bile-induced protein aggregation (Lee et al., 2020). UgpB has, as a structural characteristic of periplasmic substrate-binding proteins, which are two globular domains connected by a hinge where the substrate, in this case G3P binding at the interface between the two domains (Figures 3B, 4; Wuttge et al., 2012). UgpB only exhibits chaperone activity when UgpB is in the G3P-free open state (Lee et al., 2020). Protein structural analysis and mutational targeting of G3P-binding residues revealed to us that a deep cleft opens up in UgpB when G3P is released (Lee et al., 2020). Consequently, a number of hydrophobic residues in the core cleft region are exposed, and this region then can function as a surface for chaperone activity (Lee et al., 2020). In addition, G3P can compete for the client protein that binds to UgpB, suggesting that UgpB’s G3P-binding and chaperone activities are mutually exclusive.

How is the chaperone activity of UgpB modulated in the host? Since G3P binding to UgpB inhibits its chaperone activity, there must be a mechanism to strip G3P from UgpB to prime it as an active chaperone before bacteria reach the duodenum, where bile is secreted. Bacteria have to pass through the stomach before they reach the duodenum. Since low pH unfolds proteins, including UgpB, any G3P bound to UgpB can be removed through exposure to the low pH conditions present in the stomach (Figure 3B; Lee et al., 2020). At neutral pH levels, like those present in the duodenum, UgpB is refolded, and it can thus function there as a bile-responsive chaperone (Lee et al., 2020). At subsequent points in the digestive tract, i.e., in the jejunum and ileum, bile is diluted out and efficiently absorbed, decreasing the bile concentration (Heaton, 1969; Weski and Ehrmann, 2012). Digesting food increases the G3P concentration in the jejunum and ileum, and this induces a role reversal in UgpB, in which it regains its activity as a periplasmic G3P-binding protein (Lee et al., 2020).

Diverse periplasmic substrate-binding proteins bind to their specific substrates such as amino acids, peptides, sugars and vitamins, and deliver them to the transport protein in the inner membrane to transport them into the cytoplasm (Ames, 1986). Periplasmic substrate-binding proteins share structural similarities, including two conserved domains linked by a hinge and a substrate-binding surface located at the interface between the two domains (Figure 4; Wuttge et al., 2012). Of note, in addition to UgpB, the chaperone activities of various bacterial periplasmic substrate-binding proteins, such as maltose-binding protein (MBP), galactose-binding protein (MglB), oligopeptide-binding protein (OppA), and dipeptide-binding protein (DppA), have previously been detected (Richarme and Caldas, 1997; Lennon et al., 2015). In addition, periplasmic substrate-binding proteins are very abundant at least under some conditions as UgpB and other chaperones. For example, OppA and DppA are highly induced at the stationary phase (Sangurdekar et al., 2006). MBP and OppA are the most abundant periplasmic proteins in *E. coli* K-12 and B strains (Han et al., 2014). These results suggest that at least several periplasmic substrate-binding proteins may also play roles in periplasmic proteostasis. However, their detailed molecular mechanisms and the physiological roles associated with their chaperone activities have not yet been elucidated. *E. coli* MBP is very widely used as a fusion tag to enhance the solubility of target recombinant proteins (Riggs, 2000). How an MBP-fusion tag increases the solubility of the target recombinant proteins is still unclear, but one possible explanation is that MBP functions as a cis-acting chaperone in the context of these fusions by binding to the aggregation-prone folding intermediates of the fused protein and preventing their aggregation (Richarme and Caldas, 1997; Fox et al., 2001). Importantly, the ligand-binding cleft of MBP has a hydrophobic nature, and the substitution of certain hydrophobic residues in the cleft with charged residues dramatically reduces the solubility of proteins fused to these MBP mutants (Fox et al., 2001). The chaperone activity regions for OppA and DppA have not been precisely determined, but, interestingly, co-crystal structures of OppA and DppA with their substrate peptides have shown that the peptides are bound deep inside the cleft (Dunten and Mowbray, 1995; Sleigh et al., 1999). These results suggest that the core hydrophobic cleft region is crucial for the chaperone activities of these periplasmic substrate-binding proteins, as it seems to be for UgpB.

## Concluding Remarks

Due to high affinity and low specificity of chaperone to their client proteins, chaperone activity needs to be finely regulated. If their activity is unregulated, their high affinity for the unfolded or partial-folded states may interfere with the folding process and thus be harmful to the cell. Many cytosolic chaperones utilize ATP to modulate their activity, but periplasmic chaperones require an alternative regulatory mechanism because the periplasmic space is completely lacking in ATP. To function as a chaperone during exposure to environmental stress in the periplasm, the expression level of the stress-responsive chaperones needs to be sensitively regulated. Spy, for example, is nearly absent during normal growth, but a massive induction of Spy occurs following envelope stress. In addition to regulating the expression level, periplasmic stress-responsive chaperones have a number of novel mechanisms to directly control their chaperone activities at the protein level. Spy fine-tunes its binding affinity for client proteins to enable them to fold while bound to Spy. DegP regulates its dual function as a chaperone and protease in response to temperature changes. A few of the periplasmic chaperones (e.g., HdeA, HdeB, and UgpB) take advantage of the external environmental changes associated with natural host physiology, such as the pH decline caused by the passage of bacteria through the stomach, to regulate chaperone activity. Low pH induces partial unfolding in HdeA and HdeB, activating their chaperone activities. Neutralization allows substrate release and the refolding of both the client and the chaperone. The bile-responsive chaperone UgpB also utilizes low pH as an environmental cue to activate UgpB as a chaperone. The low pH environment in the stomach strips off the bound G3P to activate the chaperone activity of UgpB, thus enabling UgpB to suppress bile-induced protein aggregation in the duodenum.

## Author Contributions

CL conceived and wrote the manuscript. HK and KW made figures and helped in the writing of the manuscript. All authors contributed to the article and approved the submitted version.

## Conflict of Interest

The authors declare that the research was conducted in the absence of any commercial or financial relationships that could be construed as a potential conflict of interest.
